# CD147 expression was positively linked to aggressiveness and worse prognosis of gastric cancer: a meta and bioinformatics analysis

**DOI:** 10.18632/oncotarget.20089

**Published:** 2017-08-09

**Authors:** Hua-Chuan Zheng, Bao-Cheng Gong

**Affiliations:** ^1^ Department of Experimental Oncology and Animal Center, Shengjing Hospital of China Medical University, Shenyang 110004, China

**Keywords:** CD147, gastric cancer, meta analysis, bioinformatics analysis

## Abstract

CD147 (also named as Basigin or EMMPRIN) might promote cancer invasion and metastasis by inducing MMP and VEGF synthesis in tumor microenvironment. We performed a systematic meta and bioinformatics analysis through multiple online databases up to March 14, 2017. Up-regulated CD147 expression was found in gastric cancer, compared with normal mucosa (*p* < 0.05). The male patients with gastric cancer showed higher CD147 expression than the female ones (*p* < 0.0001). CD147 expression was positively correlated with tumor size, depth of invasion, lymph node metastasis, TNM staging and unfavorable prognosis of gastric cancer (*p* < 0.05). At mRNA level, *CD147* expression was higher in intestinal-type and mixed-type gastric carcinomas than normal tissues (*p* < 0.05). *CD147* mRNA expression was negatively associated with histological grading and dedifferentiation of gastric cancer (*p* < 0.05). A higher *CD147* mRNA expression was negatively correlated with overall and progression-free survival rates of all cancer patients, even stratified by clinicopathological features (*p* < 0.05). These findings indicated that CD147 expression might be employed as a potential marker to indicate gastric carcinogenesis and subsequent progression, even prognosis.

## INTRODUCTION

CD147 (Basigin, M6 and tumor cell-derived collagenase stimulatory factor) was isolated from the surface of LX-1 lung carcinoma cells. Because it might increase cancer invasion by inducing MMP synthesis in neighboring fibroblasts, endothelial and cancer cells, including MT-MMP, MMP-1, MMP-2, and MMP-3, and the endogenous activators of MMP-2, CD147 is also named as EMMPRIN, which indicates its Extracellular Matrix Metalloproteinase Inducer activity [1–2]. It is a glycosylated cell surface transmembrane protein, and has core (approx 27 kDa), highly- (HG, 45–65 kDa) and lowly-glycosylated (LG, 32–44 kDa) forms [3]. Wang et al. [4] found that inhibition of N-glycosylation increased the ubiquitination and degradation of CD147. F-Box protein FBXO22 could mediate the polyubiquitination and degradation of CD147 by interacting with CD147, and CD147 polyubiquitination by FBXO22 reversed cisplatin resistance of tumor cells [5]. Jia et al. [6] reported that CD147 deglycosylation down-regulated MMP-11 expression and the adhesive capability of murine hepatocarcinoma cells. Our previous study showed that HG form was more expressed in ovarian cancer than normal ovary, and metastatic than primary cancers. HG-CD147 expression was positively correlated with FIGO staging and dedifferentiation of ovarian cancer [7]. Its two Ig-like domains in its extracellular portion induce MMP expression, while it is also cleaved by MMPs in tumor environments [8]. Serum or urine CD147 level was higher in transitional cell carcinoma and prostate cancer than healthy control [9, 10].

Reportedly, CD147 overexpression promoted cell invasion, epithelial-to-mesenchymal transition (EMT) via MAPK/ERK pathway in colorectal cancer [11]. CD147 was a target gene of Slug in TGF-β→PI3K/Akt→GSK3β→Snail→Slug→CD147 signaling cascade, finally to cause EMT of hepatocellular carcinoma (HCC) cells [12]. Ru et al. [13] found that CD147 was involved in TGF-β-induced EMT and invasion of HCC cells. Hepatocyte-specific CD147- knockout mice decreased the susceptibility to N-nitrosodiethylamine- induced tumorigenesis by suppressing TGF-β1-CD147 signaling and inhibiting dedifferentiation of hepatocytes during tumor progression [14]. Zhou et al. [15] demonstrated that CD147 mediated the chemoresistance of breast cancer via ABCG2, which affected the cellular localization and dimerization of CD147. Lv et al. [16] reported that CD147-postive cells from breast cancer tissues and cell lines possessed stem-cell-like features, including the ability of self-renewal *in vitro*, differentiation, and tumorigenic potential *in vivo*. These findings indicate that CD147 overexpression confers cancer cells more invasive and chemoresistant, which seem stemness.

CD147 has a broad tissue distribution, but its overexpression is also seen in breast cancers, HCC, esophageal and cervical squamous cell carcinoma, genitourinary, gastric, colorectal, prostate and ovarian cancers [1, 2, 17]. Liang et al. [18] found that promoter hypomethylation of CD147 might result in the cancer-related overexpression of CD147 because more Sp1 protein bound to its promoter [18, 19]. Wang et al. [20] showed that CD147 silencing inhibited cell proliferation, invasion and increased chemosensitivity to cisplatin in SGC7901 cells *in vitro*. In our previous work, CD147 expression was found to positively correlate with tumor size, depth of invasion, lymphatic invasion, expression of ki-67, MMP-2, MMP-9 and VEGF, angiogenesis and unfavorable prognosis of gastric cancer [21]. Here, we performed a meta and bioinformatics analysis to confirm the clinicopathological and prognostic significances of CD147 expression at both protein and mRNA levels.

## RESULTS

### Characteristics of eligible studies

**Figure 1:**
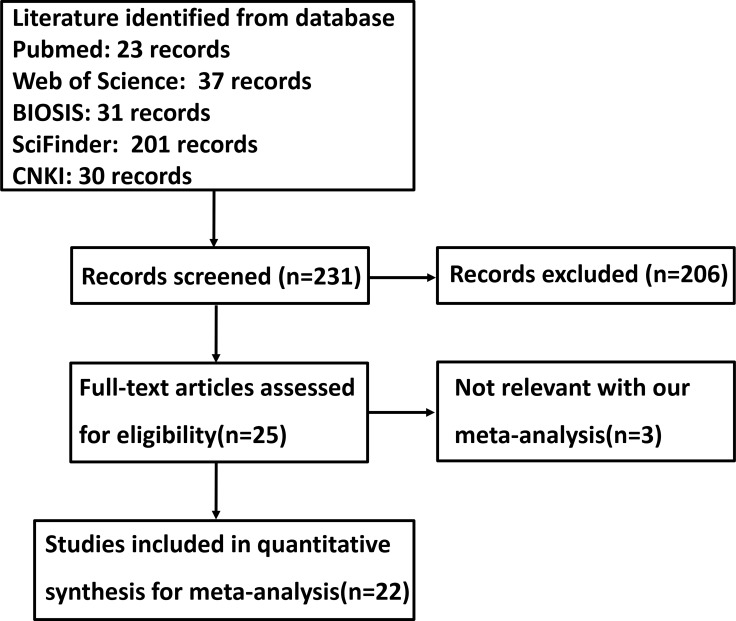
Flow diagram of the selection process in this meta-analysis

Figure 1 is a flow diagram of paper selection for our meta-analysis. As shown in Table 1, a total of 22 articles on the relationship between CD147 expression and cancer risk, clinicopathological or prognostic parameters of gastric cancer were retrieved for our meta-analysis by immunohistochemistry in PubMed, Web of Science, BIOSIS, SciFinder and CNKI. Only 20 articles contained the samples of normal gastric mucosa [21–40]. There appeared the comparison between CD147 expression and clinicopathological characteristics of gastric cancer in 22 pieces of paper, including sex, depth of invasion, lymph node metastasis, TNM staging and Lauren's classification [21–42]. Finally, we discussed the prognostic significance of CD147 expression in 4 articles [21, 24, 27, 32].

**Table 1:** Main characteristics of eligible studies

First author	Year	Country	Ethnicity	AS	Cases	Control	Risk to cancer	Outcome	Quality
Zheng HC	2006	Japan	Asian	NovoCastra	234	28	Up	Negative	9
Pinheiro C	2009	Brazil	Brazil	Zymed	160	66	---		8
Chu D	2014	China	Asian	NovoCastra	223	223	Up	Negative	9
Huang L	2015	China	Asian	Zymed	74	20	Up	Negative	9
Chen ZQ	2005	China	Asian	Zymed	40	20	Up		9
Zheng X	2005	China	Asian	Zhongshan	123				8
Huang Y	2007	China	Asian	Maxim	58	20	Up		7
Gao J	2009	China	Asian	Santa Cruz	70	5	Up		8
He C	2009	China	Asian	Maxim	50	30	Up	Negative	8
Wu Q	2010	China	Asian	Zhongshan	161	40	Up		8
Xu JM	2010	China	Asian	Santa Cruz	65	65	Up		7
Fan RG	2010	China	Asian	Zhongshan	120	120	Up		7
Liu XL	2012	China	Asian	Santa Cruz	199	21	Up		8
Liu L	2012	China	Asian	Meinuoke	441	100	Up		7
Miao J	2012	China	Asian	Zhongshan	178	10	Up		8
Chen T	2013	China	Asian	Santa Cruz	126	40	Up		7
Zhang LT	2013	China	Asian	Santa Cruz	70	20	Up		8
Zhou MX	2013	China	Asian	Maxin	70	5	Up		7
Li B	2016	China	Asian	Baijing	54	60	Up		8
Gao WH	2016	China	Asian	Zhongshan	46	40	Up	Negative	8
Zhou JF	2016	China	Asian	Baijing	40	80	Up		8

### Association between CD147 expression and cancer susceptibility of gastric mucosa

**Figure 2:**
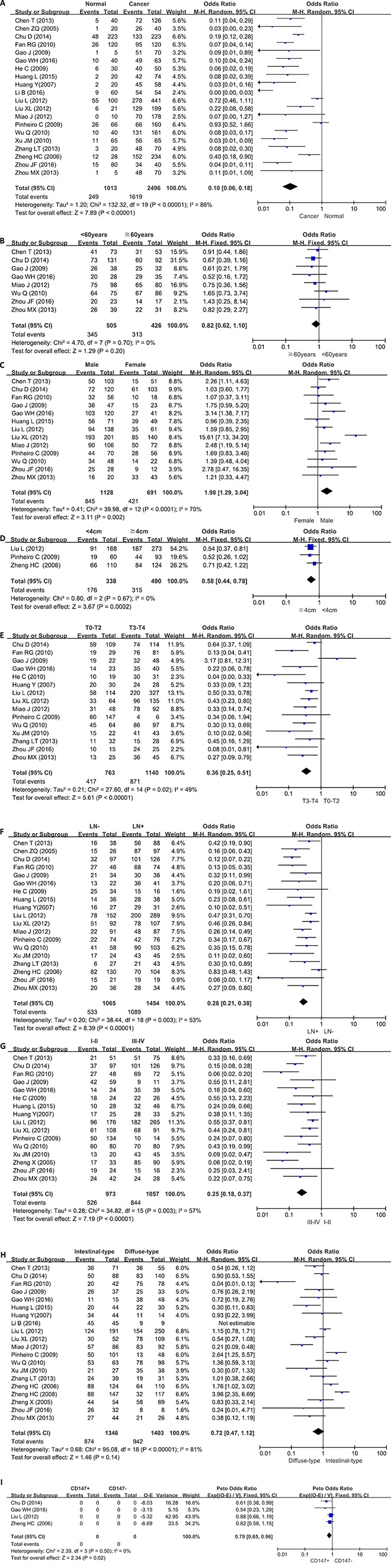
Forest plot for the relationship between CD147 expression and clinicopatholoiocal parameters of gastric cancer (**A**) gastric carcinogenesis (cancer *vs* normal mucosa); (**B**) correlation between age and CD147 expression (≧ 60 years *vs* < 60 years) ; (**C**) correlation between sex and CD147 expression (female *vs* male); (**D**) correlation between tumor size and CD147 expression (≧ 4cm *vs* < 4 cm); (**E**) correlation between depth of invasion and CD147 expression (T3-4 *vs* Tis-2); (**F**) correlation between lymph node metastasis (LN) and CD147 expression (LN+ *vs* LN-); (**G**) correlation between TNM staging and CD147 expression (stage III-IV *vs* 0-II); (**H**) correlation between differentiation and CD147 (diffuse-type *vs* intestinal-type). (**I**) correlation between survival rate and CD147 expression (CD147 − *vs* CD147 +).

We analyzed the association between CD147 expression and cancer susceptibility of gastric normal mucosa in 20 studies with 2496 cancers and 1013 controls. As a result, we found up-regulated CD147 expression in gastric cancer, compared with normal mucosa (Figure 2A, *p* = 0.002).

### Association between CD147 expression and clinicopathological parameters of gastric cancer

As shown in Figure 2B, there was no difference in CD147 expression between younger (< 60 years) and elder (≧ 60 years) patients with gastric cancer (*p* > 0.05). The male patients with gastric cancer showed higher CD147 expression than the female ones (Figure 2C, *p* < 0.0001). The large cancers (≧ 4 cm) displayed more CD147 expression than the small ones (< 4 cm, Figure 2D, *p* < 0.001). A lower CD147 expression was detected in Tis-2 than T3–4 gastric cancers (Figure 2E, *p* < 0.00001). CD147 expression was positively related to lymph node metastasis of gastric cancer (Figure 2F, *p* < 0.00001). Gastric cancers with stage III-IV showed CD147 overexpression, compared with ones with stage I-II (Figure 2G, *p* < 0.00001). There was no difference in CD147 protein expression between intestinal-type than diffuse-type carcinomas (Figure 2H, *p* > 0.05).

### Association between CD147 expression and survival rate of gastric cancer

As indicated in Figure 2I, the pooled result from 4 studies demonstrated a significant association between CD147 expression and unfavorable overall survival in patients with gastric cancer (HR = 0.79, 95% CI:0.65–0.96, *p* < 0.05). Results showed that CD147 overexpression had an unfavorable prognostic value in gastric cancer patients.

### Publication bias

**Figure 3:**
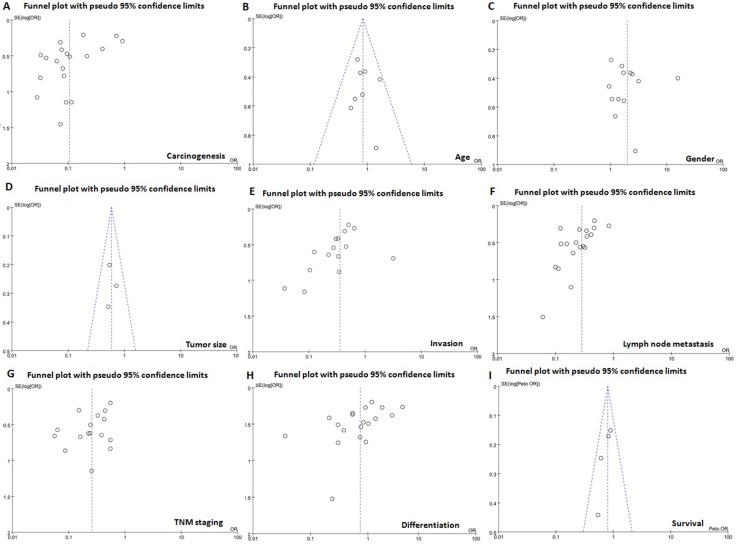
Funnel plot for publication bias test between CD147 expression and gastric carcinogenesis or progression The bias was analyzed about risk degrees of CD147 expression in gastric mucosa (**A**) for gastric carcinogenesis. Additionally, it was tested between CD147 expression and clinicopathlogical features of gastric cancer, including age (**B**), gender (**C**), tumor size (**D**), depth of invasion (**E**), lymph node metastasis (**F**), TNM staging (**G**), and differentiation (**H**) and prognosis (**I**).

The heterogeneity test was performed as shown in Figure 3. Sensitivity analysis was used to evaluate individual study's influence on the pooled results by deleting one single study each time from pooled analysis. The T-staging result of CD147 expression in Gao's study had a significant effect on the pooled OR. When this study was excluded, the heterogeneity test was significantly reduced (data not shown).

### The clinicopathological and prognostic significances of *CD147* mRNA expression in gastric cancers

**Figure 4:**
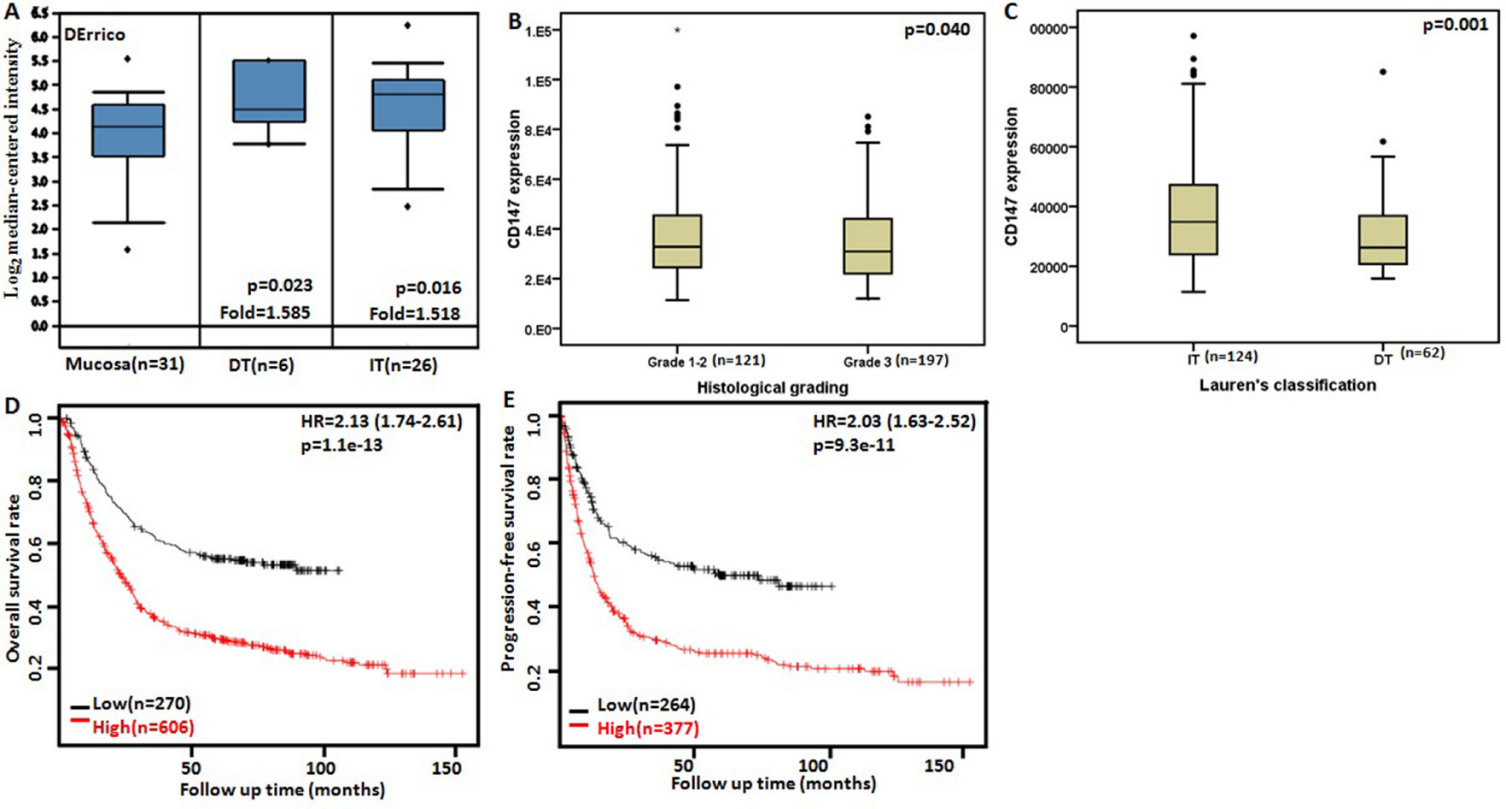
The clinicopathological significance of *CD147* mRNA expression in gastric cancer Derrico datasets were employed for bioinformatics analysis to analyze *CD147* mRNA expression during gastric carcinogenesis. A higher *CD147* expression was detectable in gastric cancer than that in normal gastric mucosa, even stratified into intestinal-type (IT) and diffuse-type (DT) carcinomas by Lauren's classification (**A**) *p* < 0.05). TCGA database shows that *CD147* mRNA expression was histologically more expressed in high-grade carcinomas than low-grade ones (**B**) *p* < 0.05). Additionally, *CD147* mRNA over expression was observed in IT carcinomas in comparison to DT ones (**C**) *p* < 0.05). According to the data from KM plotter, *CD147* mRNA expression was positively related to both overall (**D**) and progression-free (**E**) survival rates of the patients with gastric cancer. HR, hazard ratio.

Then, we used DErrico's dataset to perform bioinformatics analysis and found that *CD147* mRNA expression was higher in intestinal-type and diffuse-type gastric carcinomas than normal tissues (Figure 4A, *p* < 0.05). According to TCGA data, *CD147* mRNA expression was higher in Grade 1–2 than Grade 3 carcinomas (Figure 4B, *p* < 0.05). It was more expressed in intestinal-type than diffuse-type carcinomas (Figure 4C, *p* < 0.05).

According to Kaplan-Meier plotter, we found that a higher *CD147* mRNA expression was negatively correlated with overall and progression-free survival rates of all cancer patients (Figure 4D and 4E, p < 0.05). As shown in Table 2, it was the same for the patients with any gender, stage II-IV, T2–3, N1–3, M0, surgery alone, 5-FU-based adjuvant, intestinal-type and diffuse-type, Her2-positive and -negative carcinomas (*p* < 0.05).

**Table 2:** The prognostic significance of *CD147* mRNA in gastric cancer

Clinicopathological features	Overall survival		Progression-free survival	
	Hazard ratio	*p*	Hazard ratio	*p*
Sex				
Female	1.88 (1.29 − 2.75)	8e−04	1.62 (1.1 − 2.4)	0.014
Male	2.32 (1.81 − 2.99)	1.4e−11	2.31 (1.77 − 3.01)	2.9e−10
TNM staging				
I	3.15 (0.87 − 11.42)	0.066	2.06 (0.53 − 7.95)	0.29
II	2.22 (1.2 − 4.12)	0.0091	2.26 (1.21 − 4.22)	0.0084
III	2.2 (1.54 − 3.13)	8e−06	1.9 (1.3 − 2.77)	0.00068
IV	2.55 (1.03 − 6.35)	0.037	2.66 (1.17 − 6.04)	0.015
T				
2	1.79 (1.16 − 2.75)	0.0072	1.74 (1.15 − 2.62)	0.0081
3	1.83 (1.29 − 2.59)	0.00057	1.5 (1.07 − 2.1)	0.018
4	2.55 (1.03 − 6.35)	0.037	2.66 (1.17 − 6.04)	0.015
N				
0	1.61 (0.63 − 4.15)	0.32	1.5 (0.59 − 3.84)	0.39
1–3	2.08 (1.6 − 2.71)	2.7e−08	1.82 (1.41 − 2.34)	3.1e−06
1	2.58 (1.69 − 3.93)	4.9e−06	2.46 (1.61 − 3.75)	1.5e−05
2	3.13 (1.97 − 4.98)	4.1e−07	2.5 (1.61 − 3.89)	2.5e−05
3	0.52 (0.29 − 0.93)	0.024	0.52 (0.29 − 0.93)	0.026
M				
0	1.93 (1.44 − 2.57)	5.7e−06	1.65 (1.26 − 2.17)	0.00026
1	2.66 (1.41 − 5.02)	0.0017	1.64 (0.88 − 3.06)	0.12
Perforation				
−	1.43 (0.96 − 2.14)	0.076	1.36 (0.92 − 1.99)	0.12
Treatment				
Surgery alone	1.38 (1.02 − 1.87)	0.039	0.73 (0.54 − 1)	0.046
5-FU-based adjuvant	0.53 (0.36 − 0.78)	0.00097	0.51 (0.34 − 0.77)	0.00096
Other adjuvant	0.34 (0.14 − 0.84)	0.014	0.45 (0.2 − 1.05)	0.059
Differentiation				
Well-differentiated	0.36 (0.15 − 0.86)	0.017	-	-
Moderately-differentiated	1.45 (0.76 − 2.78)	0.26	0.72 (0.38 − 1.36)	0.31
Poorly-differentiated	1.34 (0.9 − 2)	0.15	1.59 (0.99 − 2.55)	0.051
Lauren's classification				
Intestinal-type	2.76 (1.96 − 3.9)	1.5e−09	2.32 (1.58 − 3.41)	1e−05
Diffuse-type	1.5 (1.04 − 2.15)	0.028	1.55 (1.08 − 2.22)	0.017
Mixed-type	1.85 (0.65 − 5.24)	0.24	0.4 (0.13 − 1.2)	0.091
Her2 positivity				
−	1.91 (1.5 − 2.43)	1.1e−07	1.82 (1.4 − 2.37)	6.3e−06
+	1.5 (1.1 − 2.05)	0.011	1.85 (1.23 − 2.77)	0.0027

## DISCUSSION

Invasion and metastasis are key and characteristic events in the aggressive biology of cancer, and become major obstacles to the treatment of malignancies. Various evidences showed that CD147 might enhance the migration and metastasis of cancer via Annexin A2/DOCK3-β-catenin- WAVE2, EGFR-src-Rac1-pSTAT3-DOCK8, integrin-FAK-PI3K/PIP3-Rac1-WAVE2 and FAK- PI3K/PIP3-Rac1-WAVE2 pathways [43–46]. Sidhu et al. [47] reported that a higher CD147 expression in lung cancer epithelial cells activated β-catenin signaling pathway, and CD147 silencing inhibited β-catenin signaling, cell migration, proliferation, anchorage- independent growth and tumor growth in a mouse tumor xenograft model. Besides, CD147 enhanced tumor growth of melanoma by up-regulating GLUT-1 level via activating PI3K/Akt signaling and increasing glucose uptake, stimulated hepatoma cells escaping from immune surveillance of T cells by interaction with Cyclophilin A, reprogrammed fatty acid metabolism in HCC cells through Akt/mTOR/SREBP1c and p38/PPARα pathways, promoted autophagy through PI3K/Akt/mTOR pathway in human prostate cancer cells, and chemosensitivity in head and neck squamous carcinoma cells by activating MAPK/ERK pathway [48–52].

In the present study, CD147 overexpression was found in gastric cancer, and positively correlated with tumor size, depth of invasion, lymph node metastasis, or TNM staging, in line with the results form bladder urothelial carcinoma, laryngeal carcinoma, thyroid cancer, colorectal cancer, ovarian cancer, glioma, and tongue squamous cell carcinoma [53–59]. These findings suggested that its up-regulation contributed to carcinogenesis and subsequent progression, and might be employed as a good marker for carcinogenesis and aggressive behaviors. A higher *CD147* mRNA in intestinal-type or G1–2 carcinoma was noted than in diffuse-type or G3 ones respectively, indicating that its distinct expression might underlie the molecular basis of differentiation in gastric cancer. In contrast, Zhu et al. [60] found that higher CD147 expression was correlated with the poor tumor differentiation of HCC. However, a high heterogeneity was seen in the correlation of CD147 expression with carcinogenesis, invasion, lymph node metastasis, TNM staging and differentiation of gastric cancer, which might be due to the various anti-CD147 antibody sources, different populations, sample selection bias, different evaluation and statistical methods, and something else.

A body evidences showed that CD147 expression was positively related to the poor prognosis of the patients with urothelial carcinoma, pancreatic cancer, tongue squamous cell carcinoma, cervical squamous cell carcinoma, laryngeal carcinoma, and advanced renal cell carcinoma [53, 54, 61–64]. CD147 expression might be demonstrated to indicate the worse prognosis of HCC, thyroid cancer, glioma and esophageal squamous carcinoma as an independent factor [55, 58, 65, 66]. Xu et al. [67] demonstrated that the patients with high CD147 expression and membranous localization predicted poor prognosis in both squamous cell carcinoma and adenocarcinoma. Here, meta-analysis showed that CD147 expression was positively linked to the worse prognosis of the patients with gastric cancer. Bioinformatics analysis indicated that *CD147* mRNA expression was negatively associated with overall and progression-free survival rates of the patients with gastric cancer, even stratified by clinicopathological features. Taken together, CD147 expression might be considered as a potential marker for the prognosis of the patients with gastric cancer at either mRNA or protein level.

Recently, Hu et al. [68] has reported that CD147 overexpression may serve as a promising diagnostic and prognostic marker for gastric cancer using a meta- and immunohistochemical study. The differences between our and Hu's studies are summarized as follows: 1) We have clarified the clinicopathological and prognostic significances of CD147 protein expression as previously reported [21]. 2) We collected more articles from Pubmed, Web of Science, Biosis, Scifinder and CNKI than Hu et al. (22 *vs* 17); 3) The prognostic significance of CD147 protein expression was analyzed by extracting the survival data using Engauge Digitizer software for our meta-analysis. 4) Importantly, we performed bioinformatics analysis to explore the clinicoapthological and prognostic significances of *CD147* mRNA expression using Oncomine, TCGA and KM plotter.

In conclusion, CD147 expression was up-regulated in gastric cancer, and positively correlated with advanced clinicopathological features and worse prognosis at both mRNA and protein levels. It might be employed as a good potential marker for carcinogenesis, aggressive behaviors and unfavorable prognosis of gastric cancer patients. However, the following limitations should be noted: (1) the potential publication bias stems from published results being predominantly positive; (2) patient populations in our study were limited, as patients came only from Asia and Brazil; (3) all of the survival data were extracted from survival curves, which may introduce subjective bias.

## MATERIALS AND METHODS

### Identification of eligible studies and data extraction

We performed a publication search using PubMed, Web of Science, BIOSIS and SciFinder updated on March 14, 2017. The following search terms were used: (CD147 OR EMMPRIN OR Basigin) AND (gastric OR stomach) AND (cancer OR carcinoma OR adenocarcinoma). Searching was done without restriction on language or publication years. Inclusion criteria for studies: (1) articles to observe the alteration in CD147 expression in gastric cancer by immunohistochemistry; (2) papers to compare CD147 expression with pathobiological behaviors and prognosis of gastric cancer by immunohistochemistry. Exclusion criteria included: (1) abstract, comment, review and meeting; (2) duplication of the previous publications; (3) Western blot, RT-PCR, cDNA microarray, or transcriptomic sequencing for CD147 expression; (4) lack of sufficient information.

### Data extraction

Based on the inclusion criteria, two reviewers (HC Zheng and BC Gong) independently extracted information from all eligible publications. The following information were included in each study: name of first author, year of publication, country, ethnicity, antibody company, numbers of cases and controls, expression alteration, and follow-up outcome. Regarding survival analysis, we used Engauge Digitizer software to extract data from Kaplan-Meier curves and calculated the Hazard ratios (HR) and their corresponding 95% confidence intervals (CI). Any disagreement was resolved through discussion until the two reviewers reached a consensus.

### Quality score assessment

Two reviewers (HC Zheng and BC Gong) independently assessed the quality of the included studies according to Newcastle Ottawa Scale (NOS) (http://www.ohri.ca/programs/clinical_epidemiology/oxford.htm). The scale consists of three components related to sample selection, comparability and ascertainment of outcome.

### Bioinformatics analysis

The individual gene expression level of *CD147* was analyzed using Oncomine (www.oncomine.org), a cancer microarray database and web-based data mining platform for a new discovery from genome-wide expression analyses. We compared the differences in *CD147* mRNA level between gastric mucosa and cancer. All data were log-transformed, median centered per array, and standard deviation normalized to one per array. The expression (RNA-seqV2) and clinicopathological data of 392 gastric cancer patients were downloaded from the Cancer Genome Atlas (TCGA) database by TCGA-assembler in R software. We integrated the raw data, analyzed *CD147* mRNA expression in gastric cancer, and compared it with clinicopathological and prognostic data of the patients with gastric cancer. Additionally, the prognostic significance of *CD147* mRNA was also analyzed using Kaplan-Meier plotter (http://kmplot.com).

### Statistics analysis

HWE was evaluated using Chi-square test in control groups of each study. Strength of association between CD147 expression and cancer risk was assessed by odds ratios with 95% confidence intervals. Statistical significance of the pooled OR was determined by *Z* test. If there was no significant heterogeneity, the fixed effect model (Mantel-Haenszel method) would be employed. Otherwise, the random effect model (DerSimonian and Laird method) would be used excluding prognostic analysis. Heterogeneity effect was then quantified by *I*^2^ test, which was subdivided into low, moderate and high degrees of heterogeneity according to the cut-off values of 25%, 50% and 75% respectively. Publication bias was evaluated by funnel plot and quantified by Begg's test and Egger's test to assess funnel plot asymmetry. Meta-analyses were performed with Revman software 5.3 and data from TCGA database was dealt with SPSS 10.0 software using student *t* test. *Kaplan-Meier* survival plots were generated and comparisons between survival curves were made with the log-rank statistic. Two-sided *p* < 0.05 was considered as statistically significant.
